# A Comparison of Harwell & FWT Alanine Temperature Coefficients from 25 °C to 80 °C

**DOI:** 10.6028/jres.117.007

**Published:** 2012-05-02

**Authors:** M. F. Desrosiers, A. M. Forney, J. M. Puhl

**Affiliations:** National Institute of Standards and Technology, Gaithersburg, MD 20899

**Keywords:** Alanine, dosimeter, dosimetry, gamma ray, irradiation temperature, temperature coefficient

## Abstract

The dosimeters used to monitor industrial irradiation processing commonly experience significant temperature rises that must be considered in the dose analysis stage. The irradiation-temperature coefficient for a dosimetry system is derived from the dosimeter’s radiation response to the absorbed dose and the irradiation temperature. This temperature coefficient is typically expressed in percent change per degree. The temperature rise in dosimeters irradiated with high-intensity ionizing radiation sources can be appreciable. This is especially true for electron-beam processing in which dosimeter temperatures can approach 80 °C. A recent National Institute of Standards and Technology (NIST) study revealed modest (0.5 % to 1.0 %) deviations from the predicted value at temperatures above 70 °C for absorbed doses of 1 kGy and 20 kGy. However, these data were inconsistent with a concurrent manuscript published by National Physical Laboratory (NPL) researchers that found a significant dose-dependent non-linear alanine response but used dosimeters from a different manufacturer and a different experimental design. The current work was undertaken to reconcile the two studies. Alanine dosimeters from each manufacturer used by NIST and NPL were co-irradiated over a wide range of absorbed dose and irradiation temperature. It was found that though there was a slight variation in the temperature coefficient between the two alanine dosimeter sources both systems were linear with irradiation temperature up to 70 °C and the NPL observations of non-linearity were not reproduced. These data confirmed that there is no fundamental difference in the two commercial alanine dosimeter sources and that temperature corrections could be made on industrial irradiations at the extremes of irradiation temperature and absorbed dose.

## 1. Introduction

The accuracy of the absorbed dose measurement in industrial irradiation processing dosimetry can be improved by adjusting the response of a dosimeter for the average temperature during irradiation [1]. The temperature coefficient, or relative response change per degree, is used to adjust a dosimeter’s response to the irradiation temperature employed for the system calibration. For alanine dosimeters the temperature coefficient is relatively small (approximately a tenth of a percent per degree) which helps maintain a low uncertainty despite the lack of a suitable device to accurately measure the dosimeter temperature during irradiation.

Alanine dosimeter temperature coefficients have been measured and tabulated for the multitude of early non-commercial dosimeter formulations [2,3]. Currently, two mass-produced commercial alanine dosimeters are used worldwide. They are distributed by Harwell Dosimeters Ltd. and Far West Technology (FWT).^1^ Industrial users and National Measurement Institute (NMI) transfer dosimetry services rely on these two manufacturers for their alanine dosimeters. A recent temperature coefficient study of FWT alanine dosimeters focused on irradiation temperatures in excess of 50 °C measured a temperature coefficient of 0.12 %/K at 1 kGy and a temperature coefficient of 0.10 %/K at 20 kGy that was linear up to 70 °C [4]. The finding that the temperature coefficient for 20 kGy is less than for 1 kGy is consistent with previous findings for other alanine dosimeters [2]. The reduction in the temperature coefficient for doses up to 20 kGy compared to 1 kGy (and 100 kGy) has now been documented for three different alanine dosimeter formulations by Nagy *et al.* [2], and is likely a characteristic trait of the L-alanine dosimeter system. The dosimeter response plotted versus the irradiation temperature deviates from linearity at 75 °C and 80 °C for both 1 kGy and 20 kGy [4]. For an absorbed dose of 1 kGy the dosimeter response at 75 °C is approximately 4 % lower than that predicted by the temperature coefficient, and the measured value at 80 °C is approximately 12 % lower [4]. At 20 kGy the measured value is approximately 5 % and 6 % lower than that predicted for 75 °C and 80 °C, respectively [4]. Very different findings were reported for a temperature coefficient study of the Harwell alanine dosimeter by the NPL [5]. The effect of irradiation temperature on the response of Harwell alanine dosimeters begins to deviate from linearity at temperatures above 50 °C [5]. At high doses and temperatures the effect is dose dependent and results in a change from a positive to negative temperature coefficient [5].

The key contrasting results from the NIST [4] and NPL [5] studies are:

1. The NPL/Harwell TC curves are stated to “deviate significantly from linearity” above 50 °C [5]. The NIST/FWT curves are linear to 70 °C [4].
2. The NPL/Harwell data display significant dose dependence for the TC values and TC curve shapes. The differences with absorbed dose for the NIST/FWT TC curves are relatively modest and consistent with previously published trends [2].
3. Negative slope and TC values are reported at high temperatures at 32 kGy and 70 kGy for the NPL/Harwell study [5]. The TC curve slopes are positive up to 70 °C for the NIST/FWT study; a positive slope is consistent with previous studies for other alanine systems [6,7].

Unfortunately, the NIST and NPL findings cannot be reconciled from these recently published data. The only previous publication to directly compare Harwell and FWT dosimeters did conclude their respective temperature coefficients were equivalent but was limited to a maximum dose of 50 kGy and maximum irradiation temperature of 50 °C [8]. That work measured temperature coefficients of 0.11 %/K and 0.10 %/K for Harwell and FWT dosimeters respectively [8].

There are significant differences in the most recent NIST and NPL studies, the most obvious being that the dosimeters are manufactured with different formulations by different companies. Furthermore, the absorbed doses for the study are not equivalent and the irradiation conditions (geometry, temperature control, and equilibrium material) differ. There are slight differences in the alanine dosimeter response protocol that may or may not be relevant; NPL normalizes the test measurement to a reference alanine dosimeter that requires dosimeter removal/replacement for comparative measurement whereas NIST employs an *in situ* synthetic ruby EPR reference that is measured in tandem with the test alanine dosimeter in place during the entire measurement cycle [9].

This work intends to address the following questions:

- Are the NPL/NIST differences due to the source of the alanine dosimeters?
- Are the two commercial dosimeters fundamentally different in their response to temperature?
- Will the FWT system yield results similar to Harwell alanine at higher doses?
- Can the source(s) of the NPL/NIST temperature coefficient discrepancy be identified?

## 2. Materials and Methods

The irradiations for this study were performed using a Gammacell 220 ^60^Co irradiator (serial number 207; Nordion, Canada) with a dose rate of 11.1 kGy/h. The calibration scheme for determining the dose rate was first described in NIST SP250-45 and later revised [11].

A custom aluminum rod holder, previously described by Desrosiers et al. [4], was used to achieve and maintain thermal equilibrium during irradiation. Alanine pellet dosimeters from two manufacturers were irradiated simultaneously while stacked inside the hollowed-out center of the aluminum rod. Three dosimeters of type FWT-50-10 Lot T030901 were alternated in a vertical stack with three dosimeters of Harwell Radspin Lot AF583. At the top and bottom of the stack, a dummy pellet was inserted so that all test dosimeters would be in contact with an equal surface area of aluminum. The aluminum stem containing the stack of eight dosimeters was then placed inside a stainless-steel dewar and covered with a cork lid. The lid had a hole in the center that allowed the air hose to be pointed directly over the aluminum stem. The experimental design was modified for irradiations in polystyrene; dosimeters were placed in a polystyrene stem substituted for the aluminum stem.

Temperature during the irradiation was controlled by using a high-flow air shower from a TurboJet (FTS Systems) and measured every 60 s with a type-T thermocouple. Each irradiation was preceded by an equilibration period of 20 minutes to 60 minutes of temperature stability. At the conclusion of the irradiation the dosimeters were reintroduced to the room-temperature environment.

Forty-two irradiations were performed in total. For the aluminum stem assembly, five dose levels (1 kGy, 7 kGy, 15 kGy, 32 kGy, and 70 kGy) were each processed using six temperatures (25 °C, 40 °C, 50 °C, 60 °C, 70 °C, and 80 °C). The polystyrene stem assembly was used for two dose levels (7 kGy and 32 kGy) and the same six temperatures.

The dosimeters were measured within 24 h to 48 h postirradiation with a Bruker EPR spectrometer, model ECS106. The EPR response is corrected for dosimeter mass and is normalized to an *in situ* ruby reference standard [9]. The EPR measurement parameters for alanine were: center field, 345.5 mT; microwave power, 0.5 mW; magnetic-field sweep width, 1.0 mT; modulation amplitude, 0.285 mT; time constant, 1.3 s. The measurement uncertainty for the EPR data represented in this work is 0.4 %.

## 3. Results

To resolve the questions of whether differences exist for the irradiation temperature influence on the two commercial dosimeters, FWT and Harwell alanine dosimeters were co-irradiated under identical conditions. The first dose level chosen for this study was 1 kGy. This dose level is of value to the study despite being relatively low for the high-dose, high-temperature issues under investigation here. These data are important from an EPR measurement perspective as the alanine system has been shown to respond most consistently at 1 kGy compared to doses in the tens of kGy. For example, the post-irradiation response of the alanine system is most stable at 1 kGy or less [12] and the dose rate effect on the alanine response is nonexistent or insignificant at 1 kGy but measurable at 10 kGy and up [11]. It has been postulated that the alanine response may be more influenced by the presence of secondary free radicals in the dose ranges above several kGy [11,13]. Therefore, the study began at the 1 kGy level where the alanine dosimeter response can be considered less susceptible to influence quantities commonly experienced in radiation processing.

**Fig. 1:**
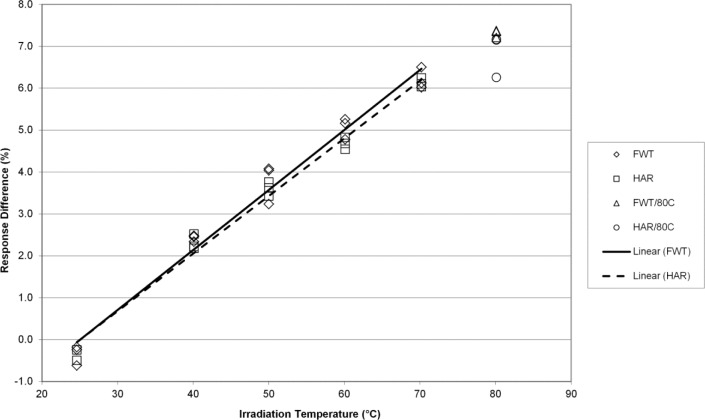
The percent change in the alanine dosimeter response from the regression-predicted value at 25 °C as a function of the irradiation temperature for 1 kGy irradiated Harwell and FWT dosimeters.

The temperature coefficients presented here are percentages of the regression slope (signal units per K) with respect to the predicted signal value at 25 °C, and are expressed in %/K. Figure 1 shows the response trends of the FWT and Harwell alanine system as a function of the average temperature during irradiation. Both systems are equivalent in terms of response over the temperature range 25 °C to 70 °C. As these data were acquired for comparison purposes to published data and to the higher doses (32 kGy and 70 kGy) studied here that are relevant to irradiation processing at high temperature, the question of linearity above 70 °C was not strictly investigated for reasons that will be apparent (*vide infra*). For 1 kGy each data set yields a temperature coefficient of 0.14 %/K. These TC’s are consistent with previously published values by both NIST [4,8] and NPL [5,14].

**Fig. 2:**
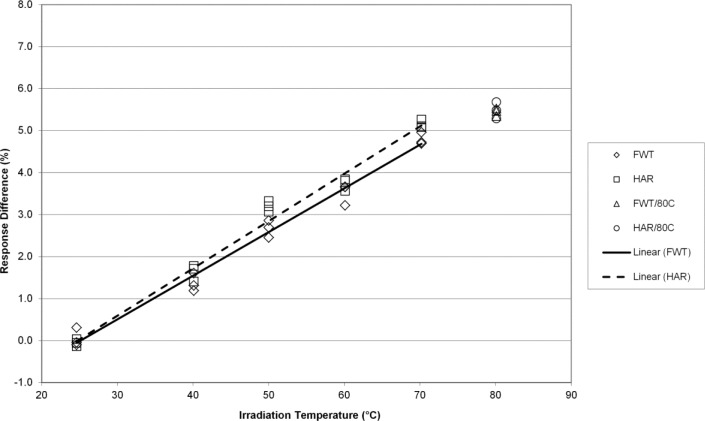
The percent change in the alanine dosimeter response from the regression-predicted value at 25 °C as a function of the irradiation temperature for 7 kGy irradiated Harwell and FWT dosimeters.

**Fig. 3:**
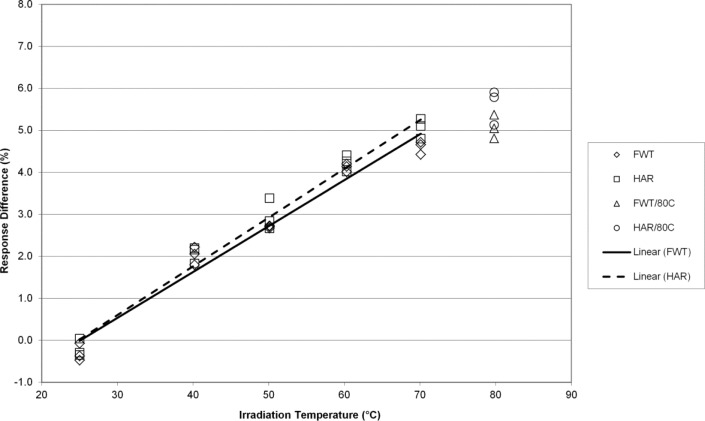
The percent change in the alanine dosimeter response from the regression-predicted value at 25 °C as a function of the irradiation temperature for 15 kGy irradiated Harwell and FWT dosimeters.

**Fig. 4:**
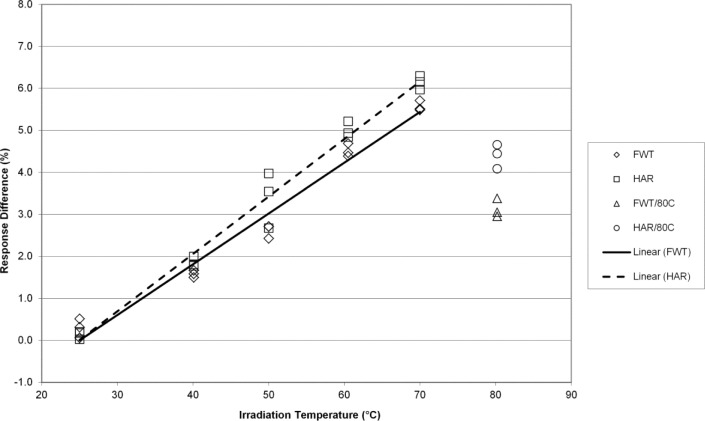
The percent change in the alanine dosimeter response from the regression-predicted value at 25 °C as a function of the irradiation temperature for 32 kGy irradiated Harwell and FWT dosimeters.

**Fig. 5:**
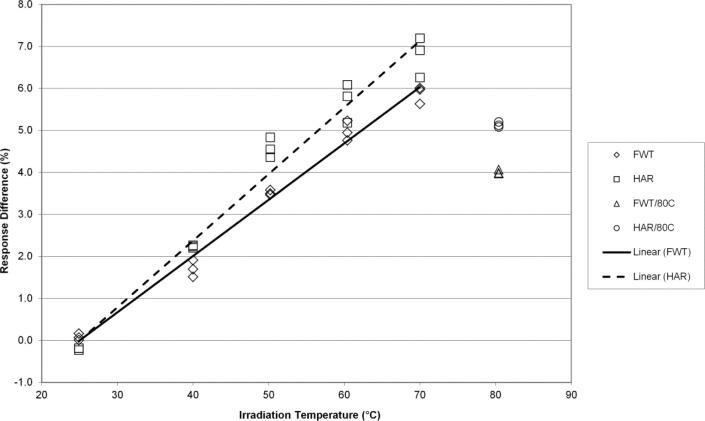
The percent change in the alanine dosimeter response from the regression-predicted value at 25 °C as a function of the irradiation temperature for 70 kGy irradiated Harwell and FWT dosimeters.

The EPR responses of the FWT and Harwell alanine dosimetry systems track well together with irradiation temperature yielding temperature coefficients that are for practical purposes equivalent at 7 kGy (Fig. 2). Figure 3 displays similar results for the FWT and Harwell alanine dosimetry systems at 15 kGy. Here again, both systems yield similar results up to 70 °C and a deviation from linearity is apparent at 80 °C. Larger differences in the temperature coefficient for the two dosimetry systems are measured at a dose of 32 kGy and the deviation from linearity is marked at 80 °C (Fig. 4). The divergence of the temperature coefficients for the FWT and Harwell alanine dosimetry systems at high dose is more apparent at 70 kGy and the deviation from linearity at 80 °C remains large for both systems (Fig. 5).

The temperature coefficients for the FWT and Harwell alanine dosimetry systems were compiled in Table 1 to emphasize the trend with absorbed dose. The temperature coefficients measured for the two systems are very similar across the entire range of doses. Note that the measured coefficient at 7 kGy is lowest for both systems. This behavior has been observed previously in two other alanine dosimetry systems [2]. Those two were a now-defunct commercial alanine dosimeter manufactured by Bruker Biospin and another custom alanine dosimeter fabricated in NIST facilities [15]. This measurement feature has now been observed in four different systems over the course of a decade. It is apparent from these data that a depression in the temperature coefficient trend over the dose range from 1 kGy up to 100 kGy is a common feature of the alanine system that is independent of the dosimeter formulation, and most likely intrinsic to L-α-alanine.

**Fig. 6:**
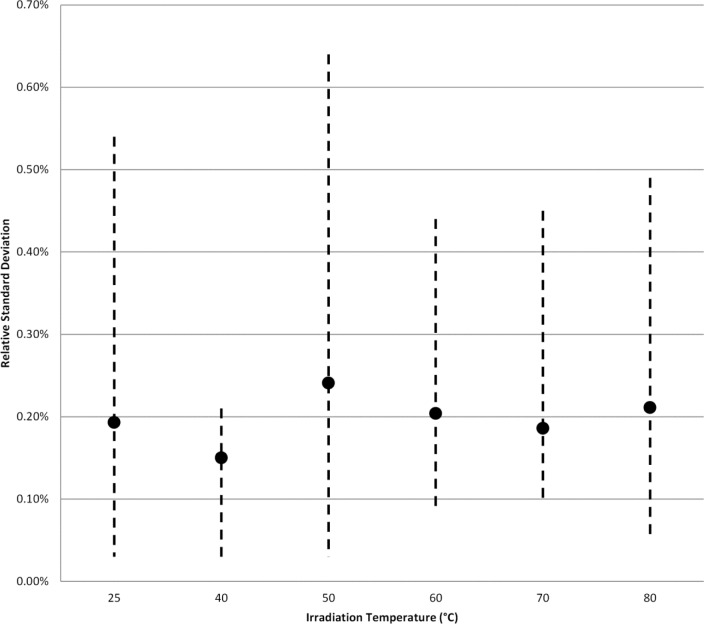
The relative standard deviation (RSD) for Harwell and FWT dosimeters for each irradiation temperature. The termini of the dashed lines represent the highest and lowest RSD measured for a group of three Harwell or FWT dosimeters at the prescribed irradiation temperature. The solid circle represents the mean value of all RSD’s measured.

**Fig. 7:**
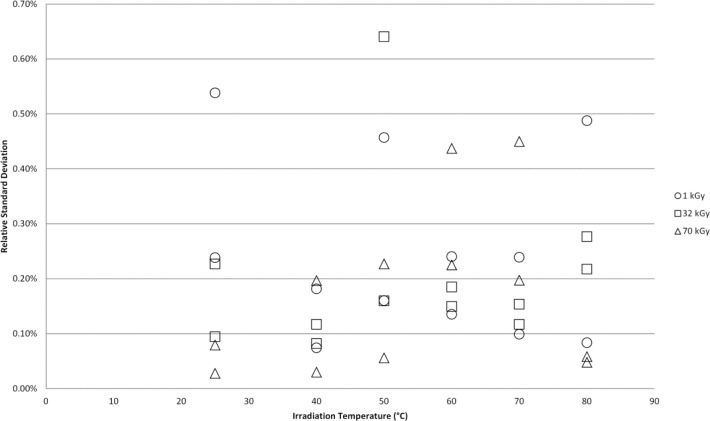
Individual relative standard deviation (RSD) values from the Fig. 6 datasets for three selected doses: 1 kGy, 32 kGy, and 70 kGy. The two data points at each irradiation temperature result from an RSD computed for the Harwell and FWT alanine dosimeter responses.

**Fig. 8:**
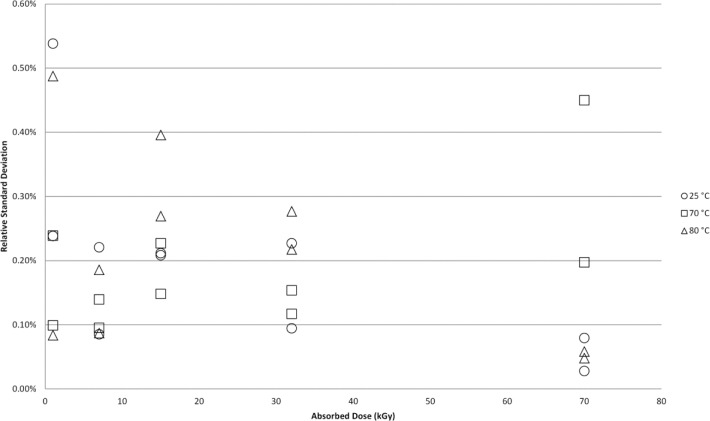
Individual relative standard deviation (RSD) values from the Fig. 6 datasets for three selected temperatures: 25 °C, 70 °C, and 80 °C. The two data points at each absorbed dose result from an RSD computed for the Harwell and FWT alanine dosimeter responses.

One of the observations of Sharpe et al. stated in the discussion of the data was that the region of high temperature above 50 °C and high dose (32 kGy and 70 kGy) “exhibits a significant increase in the scatter from replicate pellets”. To examine if a similar trend could be measured from the NIST data for Harwell dosimeters and FWT dosimeters the relative standard deviation (RSD) for each dosimeter type was determined at each dose level and associated irradiation temperature. Figure 6 shows the range of RSD’s calculated for each irradiation temperature. There are 10 RSD’s calculated for each irradiation temperature and the mean of these data are represented by the data point with each terminus of the dashed line representing the highest and lowest calculated RSD. The mean RSD is effectively constant across the entire temperature range and the range of RSD’s shows no trend towards increased scatter. Figure 7 plots some of the individual RSD values from the Fig. 6 datasets for three doses: 1 kGy, 32 kGy, and 70 kGy. The data show no trend towards increased scatter with increasing temperature for all three doses. Similarly, Fig. 8 shows no trend towards increased scatter with increasing dose from low to high irradiation temperatures.

**Fig. 9:**
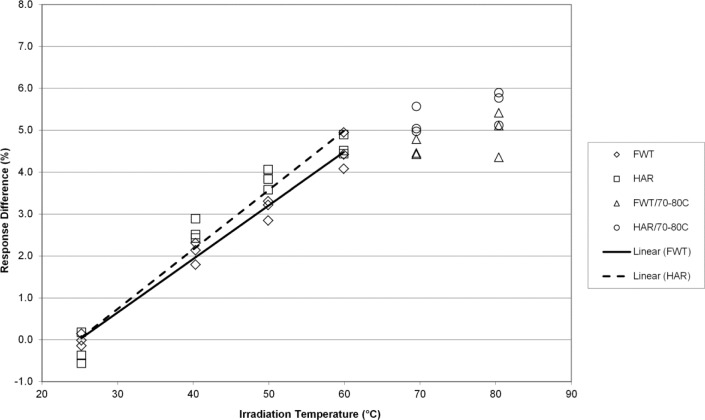
The percent change in the alanine dosimeter response from the regression-predicted value at 25 °C in the polystyrene irradiation phantom as a function of the irradiation temperature for 7 kGy irradiated Harwell and FWT dosimeters.

**Fig. 10:**
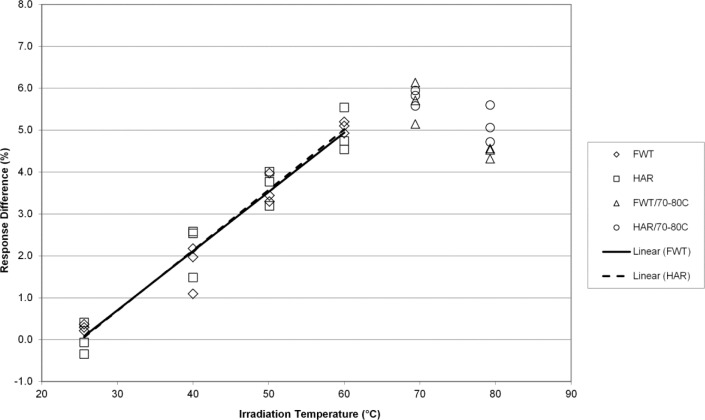
The percent change in the alanine dosimeter response from the regression-predicted value at 25 °C in the polystyrene irradiation phantom as a function of the irradiation temperature for 32 kGy irradiated Harwell and FWT dosimeters.

One of the main differences between the NIST and NPL experimental design is that the NIST irradiation geometry has the dosimeters in direct contact with the aluminum holder whereas the NPL design has the dosimeters encased in polystyrene surrounded by an aluminum housing. To examine the effects of a polystyrene holder on the NIST measurements, experiments were conducted in an analogous manner with the exception that a polystyrene dosimeter holder was used in place of the aluminum holder. Figures 9 and 10 show the response for the Harwell and FWT alanine dosimeters irradiated to 7 kGy and 32 kGy, respectively, over a range of irradiation temperatures from 25 °C to 80 °C. For most of these data the alanine dosimeter response is linear up to about 60 °C; the exception being Harwell dosimeters at 7 kGy that exhibit no linearity across the entire range of temperatures (the dashed line through these data is for illustrative purposes only). Above 60 °C and for both dose levels the response deviates from linearity for each dosimeter type. Measurements of the temperature coefficients over the temperature range from 25 °C to 60 °C for the linear data sets yielded temperature coefficients that were slightly higher in two of three cases. For these data the temperature coefficients were 0.12 %/K and 0.14 %/K for FWT at 7 kGy and 32 kGy respectively, and 0.14 %/K for Harwell at 32 kGy. The rise in response versus irradiation temperature for Harwell dosimeters at 7 kGy is non-linear but would predict a temperature coefficient greater than 0.12 %/K by comparison to the FWT data at the same dose (Fig. 9).

## 4. Conclusions

The unusual temperature and dose dependent behavior reported by Sharpe et al. [5] for the Harwell alanine dosimetry system was not replicated in the NIST study. The Harwell alanine system, measured here in conjunction with the FWT system responded in a manner consistent with the FWT system of the current study as well as other alanine dosimetry systems previously studied. In fact, aside from relatively minor differences in the individual temperature coefficient values the Harwell and FWT alanine dosimetry systems respond in an equivalent manner to the irradiation temperature. In this study,

- The alanine dosimeter response to irradiation temperature (when irradiated in the NIST aluminum holder geometry) is linear from 25 °C to 70 °C for both dosimetry systems at all dose levels from 1 kGy to 70 kGy (Figs. 1–5).
- Significant deviations from linearity are only observed at an irradiation temperature of 80 °C for both systems.
- The measured temperature coefficients are consistent with previously published values [2–5,8,14].
- Both dosimetry systems exhibit a small dose dependence in the measured temperature coefficient (Table 1). This feature may be intrinsic to alanine as it has been observed previously for two other alanine dosimeter formulations [2].

While a rigorous examination of the root cause of the unusual behavior reported by Sharpe et al. for the Harwell system is not the subject of this work, the NIST irradiation temperature studies conducted in a polystyrene irradiation phantom offer insight. Unusual temperature dependent behavior is observed when both dosimetry systems are irradiated in a polystyrene phantom under the same conditions used for the aluminum phantom (Figs. 9–10). For these data the temperature coefficients were higher and the onset of non-linearity was 10 °C lower. The implications of these observations are that the dosimeters are not in thermal equilibrium with their environment and it suggests that the internal temperature of the polystyrene phantom is higher than its immediate environment. The unusual behavior observed in the NPL study would be consistent with dosimeter irradiations at a higher temperature than that measured. Supporting evidence for inaccurate temperature control in the NPL irradiation configuration comes from their finding [5] that the measurements for replicate alanine pellets exhibits increased scatter with increasing dose and temperature; a finding that implies thermal gradients are present within the NPL polystyrene phantom. Even at extreme irradiation temperature, no increase in the measurement scatter with dose and temperature was observed for irradiations in the NIST aluminum phantom consistent with a temperature that was uniform within the irradiation volume (despite the larger volume of six pellets versus four in the NPL geometry).

In conclusion, these findings indicate that, contrary to the findings of Sharpe et al., the Harwell alanine dosimetry system should be used with the expectation that it will respond to irradiation temperature in a manner equivalent to other alanine dosimetry systems. These data are important to industrial users of the Harwell and FWT alanine dosimetry systems both for routine use and in conjunction with reference transfer dosimeters from calibration laboratories.

## Figures and Tables

**Table 1:** Alanine temperature coefficients (TC) for Harwell and FWT dosimeters measured at five absorbed doses (values are ±0.01 %/K).

Absorbed Dose (kGy)	Harwell TC (%/K)	FWT TC (%/K)
1	0.14	0.14
7	0.11	0.10
15	0.12	0.11
32	0.14	0.12
70	0.16	0.13

## Footnotes

1 Certain commercial equipment, instruments, or materials are identified in this paper to foster understanding. Such identification does not imply recommendation or endorsement by the National Institute of Standards and Technology, nor does it imply that the materials or equipment identified are necessarily the best available for the purpose.
